# Sparse outer longitudinal muscle layer in peranal endoscopic myectomy: reinforcement of wound closure with peptide gel

**DOI:** 10.1055/a-2467-3573

**Published:** 2024-11-29

**Authors:** Yoshiaki Ando, Takashi Kanesaka, Tomoki Michida, Ryu Ishihara

**Affiliations:** 1 53312 Department of Gastrointestinal Oncology, Osaka International Cancer Institute, Osaka, Japan; 2 Department of Gastroenterology and Hepatology, Osaka University Faculty of Medicine Graduate School of Medicine, Osaka, Japan

Peranal endoscopic myectomy (PAEM) is a novel endoscopic technique for removing lesions together with the inner circular muscle layer in patients with lower rectal tumors with severe submucosal fibrosis 1 2 3 4 . In this procedure, the outer longitudinal muscle is exposed at the resection area and its fragility poses a risk for perforation. As there are few reports on PAEM, the thickness of the muscles in the lower rectum is not well known. In our experience, the outer longitudinal muscle is usually dense and the extraluminal tissue is invisible ( Fig. 1 ). Herein, we present the management of a patient with a sparse outer longitudinal muscle layer ( Video 1 ).

**Fig. 1:**
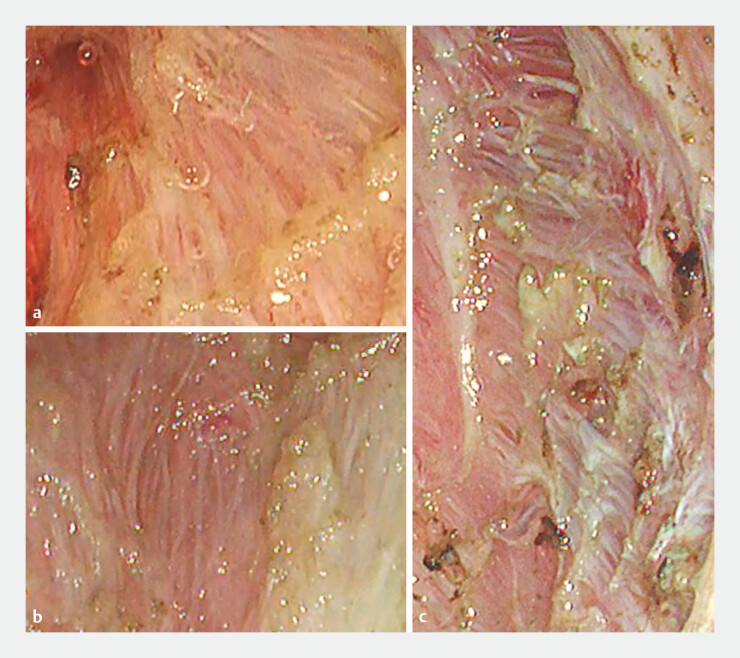
Previous cases of peranal endoscopic myectomy showing the dense outer longitudinal muscle layer at the resection margin ( **a–c** ).

Management of a sparse outer longitudinal muscle layer in peranal endoscopic myectomy.Video 1

A 52-year-old man underwent cold snare polypectomy for a small rectal lesion. Histological examination of the resected specimen revealed a 5-mm neuroendocrine tumor, grade 1, with a positive vertical margin. Additional surgery was recommended according to the Japanese guidelines 5 , and he was referred to our hospital.

No residual tumor was detected around the scar ( Fig. 2 ). After discussions with surgeons regarding the patient’s desire to preserve anal function, PAEM was performed. During the procedure, sparse and partially injured outer longitudinal muscles were observed ( Fig. 3 ). After en bloc resection, the muscle layer was roughly closed with endoclips, and a self-assembling peptide gel (PuraStat; 3D-Matrix, Tokyo, Japan) was injected into the gap ( Fig. 4 ).

**Fig. 2:**
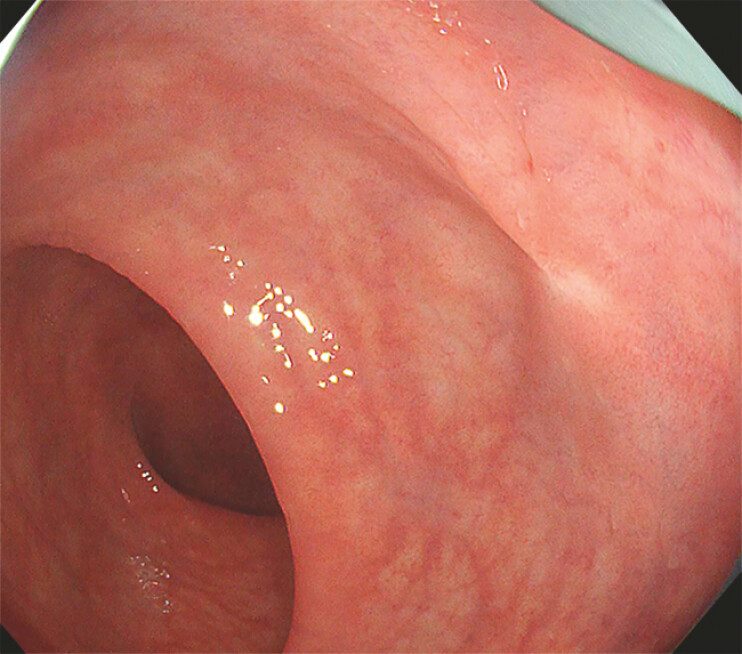
The scar after endoscopic resection for a neuroendocrine tumor in the left wall of the lower rectum.

**Fig. 3:**
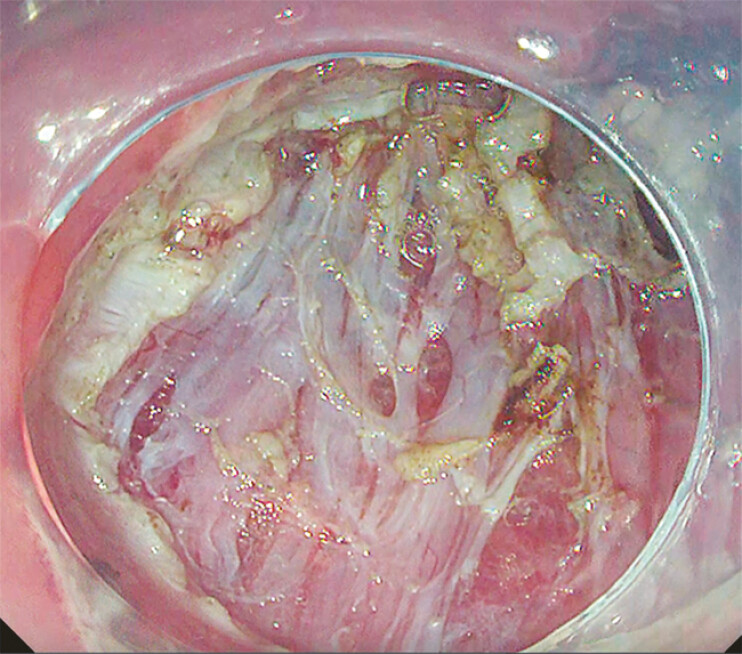
The sparse outer longitudinal muscle layer at the resection margin after peranal endoscopic myectomy.

**Fig. 4:**
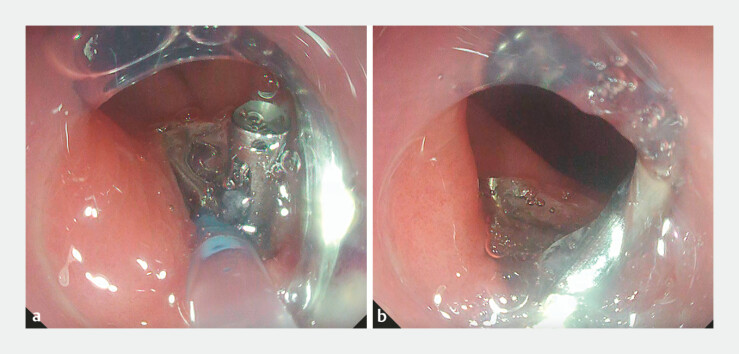
Wound closure after resection. **a** The resection wound was closed with endoclips. **b** Self-assembling peptide gel was injected into the gap.

Antibiotics were administered prophylactically on postoperative days 0 and 1. Oral intake was resumed on postoperative day 2, and the patient was discharged on postoperative day 5 without adverse events. Histological examination of the resected specimen revealed a 2-mm, grade 1 neuroendocrine tumor with a negative margin.

Injection of self-assembling peptide gel combined with endoscopic clips can be used to easily reinforce wound closure.

Endoscopy_UCTN_Code_TTT_1AQ_2AD_3AZ

## Notes

Endoscopy E-Videos https://eref.thieme.de/e-videos
*E-Videos* is an open access online section of the journal *Endoscopy* , reporting on interesting cases and new techniques in gastroenterological endoscopy. All papers include a high-quality video and are published with a Creative Commons CC-BY license. Endoscopy E-Videos qualify for HINARI discounts and waivers and eligibility is automatically checked during the submission process. We grant 100% waivers to articles whose corresponding authors are based in Group A countries and 50% waivers to those who are based in Group B countries as classified by Research4Life (see: https://www.research4life.org/access/eligibility/ ). This section has its own submission website at https://mc.manuscriptcentral.com/e-videos .
